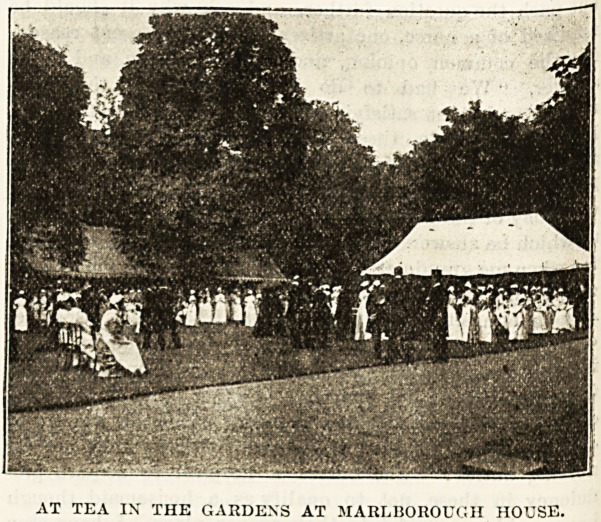# The Hospital. Nursing Section

**Published:** 1901-11-16

**Authors:** 


### The Hospital.

### Nursing Section.

Contributions for this Section of "The Hospital" should be addressed to the Editor, "The Hospital" Nursing Section, 28 & 29 Southampton Street, Strand, London, W.C.

No. 790.—Vol. XXXI. *SATURDAY, NOVEMBER 16, 1901.*

### Notes on News from the Nursing World.

### AN APPEAL BY MISS FLORENCE NIGHTINGALE.

That the British public will turn a deaf ear to the eloquent appeal made by Miss Florence Nightingale on behalf of the Home for Gentlewomen in temporary illness, we cannot for a moment believe. For the sake of people with short memories only, we recall the circumstance that even before Miss Nightingale went on her mission of mercy to the Crimea, she provided, with the assistance of Lady Canning, at 90 Harley Street, an establishment in which governesses (whose claims always have priority), the wives and daughters of the clergy, of naval, military, and other professional men, receive every possible care, comfort, and first-rate advice at the most moderate cost. The home is still conducted, as it was at the beginning, by a committee of ladies, and how greatly it is appreciated may be judged by the fact that the 20 beds are always occupied. The average cost of each patient, Miss Nightingale tells us, is £3 10s. 7d. per week, and the contributions of the patients to their board and lodging, meet half the expenses of the home. There are no doctors' fees to pay, for the eminent physicians and surgeons who attend the patients give their services. Last year 164 patients were treated, of whom 145 were cured, and 123 operations were performed. The doctors, therefore, do their full share in the good work. But the public do not do theirs. Year after year a deficit of several hundreds of pounds has been met by trenching on the small invested capital or by using legacies instead of investing them; "but," as Miss Nightingale puts it, "for 1901 there is no such help forthcoming by legacies." Let it then be forthcoming by contributions. There are many single individuals who could easily afford the £650 a year which, with the ordinary receipts of £1,770, would free the committee from all anxiety; but we should prefer to see the amount subscribed by a number. Miss Nightingale's splendid services to her country are acknowledged with enthusiasm by tens of thousands—will they not rally to her call when she writes, "I ask and pray my friends who still remember me not to let this truly sacred work languish and die for want of a little more money."

### OUR CLOTHING DISTRIBUTION.

As usual in November we are receiving urgent appeals from the matrons of hospitals for a share in our distribution of clothing at Christmas. One of our correspondents says, "The parcel you sent last year was so thoroughly appreciated, and as our needs are greater this year rather than less, I hope you will be able to help us again." The institution in question is situated in a district containing a quarter of a million of toilers. We hope to be able not only to comply with this request, but also, by the kindness of our readers, to be in a position to send a few parcels to deserving charities which we have hitherto been compelled to omit from our list.

### THE LOCAL GOVERNMENT BOARD AND CROYDON INFIRMARY.

Instead of attempting to answer the question of a correspondent who asks, not without good reason, whether the Local Government Board would recognise a certificate without the signature of the matron of Croydon Union Infirmary, we referred the matter to the Board itself. The following reply has been received from the assistant secretary at Whitehall:—"I am directed by the Local Government Board to advert to your letter of the 1st instant, in reference to the certificates of nurses trained in the infirmary of the Croydon Union, and to state that they are not prepared to advise you on the points referred to in that letter." The refusal of the Local Government Board to afford information for the guidance of intending probationers is very unsatisfactory; and in the face of that refusal we certainly should not advise any nurse to enter for training at Croydon Infirmary.

### THE WAR NURSES.

The *Canada* has again arrived at Southampton from South Africa. The following nursing sisters disembarked on Monday:—M. Brough, M. Forrest, and A. Hayhurst, A.N.S.R. (require one month's leave and return to South Africa); E. M. King (requires two months' leave); R. Kennedy, Civil Nurse (time expired, but was employed on voyage home, and wishes to return to South Africa); K. E. King, A.N.S.R., was invalided home.

### NIGHT NURSING IN AN INDIAN NATIVE HOSPITAL.

A correspondent who is travelling in India is sending us some interesting experiences of his visits to hospitals and nursing homes. His account this week of the manner in which the general hospital at Rajkot is managed, will be read by nurses at home with interest. Not the least remarkable feature is the arrangement for the night duty. Our correspondent elicited from "a charming staff of hospital assistants," who seemed most willing to afford information, that there is one bungay, one puggy, and one ward boy on duty during the night. The bungay, who belongs to an outcast class, and the ward boy may go to sleep, but the puggy, or watchman, is expected "to make regular rounds of the hospital wards and grounds and outbuildings during the night." Moved, doubtless, by a vision of the bungay and the ward boy fast asleep, and the puggy perambulating the outbuildings, at a moment when a patient required instant attention, our correspondent inquired, "And if anything is wanted in the wards?" The reply was, "One of us is called; while if there is any really urgent case in the wards, we take it in turn, of four-hour watches, to sit up in the wards the whole night through." If night nursing is not yet all that it ought to be in this country, we are a little ahead of the system which appears to be considered satisfactory in a native Indian hospital.

### THE FOREIGN SECRETARY ON TRAINED NURSES.

At the opening of the comfortable and commodious Nurses' Home in connection with the Salisbury Infirmary, the Foreign Secretary was one of the speakers. In acknowledging the vote of thanks to Lady Lansdowne for proposing the opening ceremony, Lord Lansdowne declared that the employment of properly-trained nurses had been the means of robbing the sick bed, both of the poor and the rich man, of half the terrors which used to surround it in former days. That being so, he hoped that the number of nurses whom he saw present would grow greater and greater as years went by. "He rejoiced to think that they had at last been able to take effectual steps for providing the nurses with a suitable home in the vicinity of the infirmary. He supposed there was no profession which involved a greater strain, physical and mental, upon the persons who followed it, and surely it was not too much to ask that they should do what they could to provide for them when they were off duty a house in which they could be not only comfortable, but in which their surroundings were made as bright and attractive as they could possibly be." The new home, which is a local memorial of Her late Majesty's Diamond Jubilee, and has cost considerably over £7,000 to erect, has a perfectly plain exterior, the committee having wisely refrained from spending money in ornamentation. It is connected with the main block of the infirmary by a covered passage, which has been built as a memorial of the late Miss Bonham-Carter, who for four years occupied the post of superintendent of the nurses. On the ground floor are sitting rooms for the superintendent, nurses, and probationers, a waiting room for visitors, two studies, a cloak room, with lavatories, a linen sorting room, and a box room. The nurses will still continue to have their meals in the infirmary, but a small kitchen has been provided to do any necessary cooking at the new home. On the first and second floors there are 44 bedrooms, with an adequate number of bathrooms and linen closets, and one wing on the second floor has been specially set apart for the night nurses. The whole building has been erected with a view to both bedrooms and corridors obtaining the maximum of sun and air. It is heated by means of hot water in conjunction with open fireplaces; and it is lighted throughout by the electric light.

### QUEEN'S NURSES AT READING.

The interest in the work which is being done by the Queen Victoria Institute for Nursing the Sick Poor of Reading, has been admirably stimulated by a conversazione at Reading Town Hall, to which the High Sheriff of Berkshire, Mr. Blackall Simonds, who is also chairman of the governors, invited a great number of guests, and entertained them, by the aid of his wife, in the most delightful manner. Miss Clara Goslett was to have given an address on the occasion, but the fog prevented her from keeping her engagement, and the High Sheriff himself delivered a bright little speech explaining the nature of the work of the Nursing Institute. On the following day the annual meeting took place, and the satisfactory announcement was made that the over-draft of £70 due to the bankers had been paid off, while a balance of £21 was still in hand. Another matter for congratulation is that many of the poorer people subscribed their pence. The need for further subscriptions is, however, pressing, as the patients continue to increase in number, and the nursing staff therefore requires to be augmented. We are glad to see by results that the tributes which were paid to the efforts of the nurses by several prominent personages, including doctors and clergymen, must have been thoroughly well deserved.

### THE LATE SUPERINTENDENT OF THE CORK WOMEN'S HOSPITAL.

Miss Baxter, who for 14 years acted as Lady Superintendent of the County and City of Cork Hospital for Women and Children, starts a nursing home at Harrow, under the most favourable auspices; for the Queen, whose excellent judgment is not less characteristic than her graciousness, has sent her a very kind message expressing her Majesty's sympathy in the working of her new nursing home. Prior to her departure from Cork the medical and surgical staff of the hospital presented Miss Baxter with an address, in the course of which they said: "Perhaps in no way have your great powers of organisation and personal influence been more apparent than in the training and establishment of a staff of nurses, who, for skill and efficiency in the discharge of their duties, cannot be surpassed."

### THREE NURSES FOR NINETY PATIENTS.

The plea of urgency in respect to the provision of additional accommodation for the nurses in Pontypridd Workhouse Infirmary, was raised by Dr. Howard Davies, the medical officer at the last meeting of the Board of Guardians. Dr. Davies, in fact, stated that the matter was "extremely urgent," and he gave good reason for the statement. There are, it appears, 90 beds in the infirmary, all of which are occupied every night; and there are only three trained nurses to look after the 90 patients. Yet if the guardians appointed another, there is no accommodation for her. We are glad to hear that a Local Government Board inspector was present during the discussion which arose on a proposal to adjourn the question of providing additional accommodation for the nurses until the new workhouse at Llwynypia is completed. As he concurred in the appointment of a committee of inquiry in the meantime, we presume that there will now be no unavoidable delay. But the Pontypridd Guardians should not have allowed things to come to such a pass.

### DUDLEY GUARDIANS AND THE SUPERINTENDENT.

The Dudley Board of Guardians have declined to accede to a request of Miss Peers to be reinstated as superintendent nurse at the Workhouse Infirmary. Miss Peers is not the superintendent who was lately suspended, but her predecessor, who has been nursing the troops in South Africa. We think that the guardians should throw the position open to competition, and that Miss Peers should apply in the ordinary way, if she cares to apply at all. For, until the suspension of Miss Newbury is explained, the guardians must not be surprised if few nurses of standing care to compete for the post.

### THE MATRON OF THE CEYLON NURSING ASSOCIATION.

Miss Agnes Ramsay has resigned the post of lady superintendent and secretary of the Ceylon Nursing Association. Mrs. Debenham, the honorary secretary of the Colonial Nursing Association, in expressing her regret at Miss Ramsay's retirement, says, "Your work has been most arduous, but at the same time so successful, that if it were not that your health is concerned, we should urge you to stay on at all hazards. We hear on all sides of the excellent work you have been doing, and how much the Association owes to your tact and energy." Miss Ramsay has reorganised the Hatton Home at Colombo during the period of her matronship.

### CUMBERLAND NURSING ASSOCIATION.

We regret to learn from the fourth annual report of the Cumberland Nursing Association, which was adopted at a well-attended meeting in Carlisle Town Hall, that it has not been possible, owing to lack of funds, to appoint a Queen's Nurse in any newly-formed district. The association has now in its employ 26 nurses, of whom, however, only six are fully trained. The others are village nurses, whom the association find very useful in their way. Even nine months' training at Plaistow is an improvement on the old system, when Sairey Gamp picked up her knowledge as she went along.

### THE INDISPENSABLE NURSE AT SOUTH MOLTON.

At the annual meeting of the South Molton Nursing Association, Lady Susan Fortescue gave an excellent illustration of the value of "the indispensable nurse" to the sick poor. She said:

> A few days ago I had a very interesting and gratifying account of the help your nurse had given to a Filleigh woman who had been summoned hastily to South Molton to a sudden death of a relative. She literally had to go there straight from her bed, being laid up with very bad varicose veins. The nurse happened to be about when she arrived, and inquired what was the matter, and she was most kind and helpful, looking in on her and dressing her leg two or three times a day, and finally sending her home much better than when she started, in spite of all she had been obliged to do. My friend had always been a subscriber, but now hopes to increase her subscription as a token of gratitude.

The South Molton is one of the several associations to which the Totnes Board of Guardians, by a bare majority—after three trials of strength—voted a subscription of £5. Their grudging support is in striking contrast with the generosity of Dr. Hatherly, of South Molton, who being able to appreciate the good work done by the organisation, has contributed £20 to be kept as a reserve fund.

### PROGRESS AT RUGBY.

The chairman of the Rugby District Nursing Association was able at the annual meeting to congratulate the members on the fact that the subscriptions had been augmented considerably during the year. Even apart from the proceeds of the Rugby Cycling Club fête, which amounted to no less than £50, there was a balance on the right side. The Co-operative Woman's Guild raised £11 4s. by a concert, but the most satisfactory feature is that the increase in the number of subscribers was 69. We hope this means that the railway men, who form such a large proportion of the inhabitants of Rugby, mark their appreciation of the work of the Association in a practical manner. The nurses paid upwards of 5,000 visits during the 12 months.

### TROUBLE AT BRIDGEND AGAIN.

The nurse who acts as assistant matron at Bridgend Workhouse Infirmary has sent in her resignation on the ground that she cannot "put up with the master and matron any longer." The guardians have accepted the resignation, but asked the nurse to reduce her alleged grievance into writing. It is desirable that the nature of the complaints which the nurse has to make should be clearly stated, but the guardians seem to have put the cart before the horse. Why did they not take the more prudent step of deferring their acceptance of the resignation until they were in possession of the precise reason which led her to arrive at the decision that she could not remain in their service any longer?

### AN EXPERIENCED NURSE.

A short time ago a "Queen's" nurse in the West of England was asked by a doctor to see a self-willed old woman who had a bad attack of influenza, and to try and persuade her to go to bed and be properly nursed. This she flatly declined to do, saying she would not go to bed, nor would she take a "parcel of old rubbish," meaning the medicine; but she would have a "dish of tae" when she had a mind to. Nevertheless, the nurse, fearing more serious developments, called daily and for several days. When the worst was over the old woman was left a day or two without a visit, and when nurse called again she was evidently much better and ready for a gossip. She commenced by saying: So poor Mr. R—— 'as gone, 'as 'e? Did you 'tend 'e? but no, of course you didn't! because 'e 'ad an 'experienced nurse" down from Plymouth!

### NURSES' PRIZE DISTRIBUTION AT BRISTOL HOSPITAL.

The nurses' prize distribution at Bristol General Hospital took place last month, when the prizes and medals were distributed by the chairman, who afterwards entertained the nursing staff at an "At Home." The gold medal was awarded to Nurse Hadley, the silver medal to Nurse Talbot, and certificates of merit to Nurses Bridger and Page. In physiology Nurse Just took the first prize for the first year, and Nurse Bibbing the second; and for the second year Nurse Gildford the first and Nurse Rayberry the second. In surgery for the first year Nurses Nicholls and Just gained the first and second prizes respectively, and for the second year Nurses Rayberry and Gildford. Nurse Just also won the first prize for the first year in anatomy, Nurse Holbron taking the second. For the second year Nurse Rayberry took the first prize, and Nurse Hughes the second. The prize for general excellence was obtained by Nurse Davies.

### A TYPHOID PATIENT AS STOKER.

A blacksmith who had been admitted to a small provincial hospital suffering from a mild form of typhoid fever seized the opportunity one evening during the absence of the nurse to get out of bed and make up the fire. The nurse entered during the proceeding and dismay was written upon her countenance. "All right, nurse," replied the patient, cheerfully, as he ambled back to bed, "there ain't no use you gettin' riled 'cos you find someone as knows better how to stoke a fire than you do yourself."

### Lectures to Nurses on Anatomy.

By W. Johnson Smith, F.R.C.S., Principal Medical Officer, Seamens' Hospital, Greenwich.

LECTURE IV.—THE SPINE.—(*Continued.*)

The first bone, or *atlas*, fig. 7, which projects beyond the rest of the spine on either side and resembles the capital of a pillar, is little more than a large ring of bone. It presents on each side the perforated transverse process met with in

Fig. 7.—The first Cervical Vertebra or Atlas.

A Tubercles for transverse ligament; B Superior articular processes.

the lower cervical vertebræ, and the large and distinct articular processes, but there is no spinous process, and a complete absence of body. If we endeavour to seek for this characteristic feature of all other vertebræ, we shall find on examining the second cervical bone or axis, that the body of the atlas has been detached as it were from its proper vertebra and fixed on to the body of the vertebra below. The *axis*, fig. 8, which presents most of the features of an ordinary cervical vertebra, has projecting from the upper part of its proper body, and continuous with it, a well-marked bony process, called, from its supposed resemblance to a tooth, the *odontoid process*, A, but which, when viewed in front, presents very much the shape of a bishop's mitre.

The neck of the odontoid process fits into a socket formed in front by the anterior part of the ring of the atlas and behind by a ligament stretched between the two little bony projections marked A A in fig. 7. The process thus forms a pivot around which the atlas turns, carrying with it the head as the glance is directed sideways and over the shoulder. The atlas, as Mr. Holden suggested, rotates around its own body. The nodding movement of the head is effected by the rocking of two prominent oval surfaces at the base of the skull, on the two oval cup-shaped depressions, B, on the

Fig. 8.—Second Cervical Vertebra or Axis.
A Odontoid process.

upper surface of the atlas. The transverse ligament in front of the odontoid process is a structure of great importance, as it keeps the process in place and prevents it from pressing on to the upper part of the spinal cord. Such an event which would cause instant death may occur in cases of disease of the upper end of the spinal column—the so-called atlanto-axial disease—and has resulted accidentally from lifting a child by its head.

The last of the lumbar vertebræ is supported by a large triangular bone called the *sacrum* (fig. 9), which takes an important part in the formation of the pelvic girdle, and, for this reason, is often described together with the other bones of the pelvis. It seems more convenient, however, to deal at once with this bone as it is really a direct continuation of the spine enclosing the terminal portion of the spinal cord and giving passage through large orifices resembling the true intervertebral foramina to important nerves that are distributed to the lower limbs. Much as the sacrum differs in size and shape from the vertebræ we have already studied, a close examination will suggest to us that we have suddenly come not upon a bone altogether different to other bones of the spine but rather on a collection of five vertebræ which, as in a specimen of fossilised fragments, have been welded together. Along the middle of its posterior surface we observe a row of bony projections closely resembling spinous processes, and the smooth, concave surface in front is marked by transverse ridges, showing distinctly the coalescence of five vertebral bodies. We can trace also the changed forms of transverse and articular processes. That such a resemblance is not imaginary or accidental is proved by the fact that during the first fifteen years of life the sacrum is not as in the adult subject a single mass, but is made up of five distinct bones, each of which is developed or built up on a somewhat similar plan to that of the true vertebræ.

Among many points of interests in connection with this

Fig. 9.—The Sacrum.

bone we might allude to the following:—(1) The lightness of the bone in relation to its size and massive build; (2) its marked curve from above downwards; (3) its rough and irregular posterior surface presenting above two well-marked articular processes, in its middle line three spinous processes, and on either side a vertical row of four large orifices; (4) its smooth concave surface in front presenting two lateral rows of large holes for the transmission of nerves, and the well marked transverse ridges to which allusion has been made; (5) the projecting upper margin of the anterior surface, much more marked when the bone is in its natural position, which is of great importance in obstetrical work, and is called the promontary of the sacrum; (6) the thick external or lateral surfaces each presenting in front a smooth facet covered in a recent bone by cartilage and behind an expanse of bare and rugged bone. This forms on either side the joint between the sacrum and the rest of the pelvis, and is the seat of the surgical affection known as "sacro-iliac disease."

The *coccyx*—the terminal portion of the spine—supposed to resemble the beak of a cuckoo is a row of three, four, or five very stunted vertebræ, remaining for a time, which varies in different subjects, quite distinct but sooner or later coalescing to form a single bone. This portion of the spine which is a rudimental structure and devoid of function may cause some trouble, especially to women, as it is the seat of the very troublesome nervous affection known as "coccydynia."

### Hospitals without Nurses.

By a Correspondent.

I have already described my visit to the Bhownaggree Nursing Home at Bombay. Since then I have travelled many a long day and many a hot night through lands where the gaunt finger of famine is beginning to fall. I have learned that one's idea of India being a "jungle" is sheer nonsense as to great areas of it, and that in one great province at any rate—the province of Kathiawur—the outlook from the train is chiefly a great level plain, sun-scorched and parched, covered with stunted brown grass, and with fields that ought to be green, but which are now nothing but burnt-up wastes, owing to lack of rain. I have seen scores and scores of patients of all classes of life, from a Maharajah down to an outcast "sweeper" woman, and I have visited nearly every hospital and dispensary in every town that I have stayed in. As to private nursing, I have much to say, for I have again and again longed to have a trusty English nurse in whose hands I could place patients who otherwise, in spite of all their wealth, would do no good by medicines only, but for the moment I want to give an idea of the hospitals which are worked without nurses!

At the present moment I have just come back from a long drive round Rajkot, which is the political centre of the Kathiawur province. I am sitting on the verandah of our Maharajah's bungalow (each Maharajah and Thakore in the province has a State bungalow in Rajkot, which he uses when he comes to the political centre for State purposes). The servants have placed for me here a table and a couple of brass candlesticks, and as the pleasant evening breeze plays round me and the grasshoppers chirp in their thousands in the garden below I write these notes.

No Men Admitted.

We first drove to the Rasulkhanji Hospital for Women, and found the usual tablet inscription beside the door stating that it was "the princely gift to the Rajkot Civil Station of H.H. Rasulkhanji Nawab Sahib of Junagadh," and that it cost 80,000 rupees to build.

The driver held up to me an awe-stricken finger as I bade him drive in to the gates.

"No men are allowed in this hospital," he said.

"All right," I replied. "Go ahead." And, much wondering, he went.

It was a Zenana Hospital. The lady doctor in charge was away on leave, and her locum was out, but the "charge nurse" was good enough to welcome me and show me everything except the wards. This fragile, delicate-looking little Irishwoman had come out to India in childhood and had been trained in Bombay.

"Training."

"How many patients have you?" I asked.

"About 80 out-patients per day and 23 in-patient beds," she replied.

"And your nursing staff?"

"We have no nursing staff as you understand it. We have two native women who are training here and one other who does some night duty."

"Oh, you train nurses here, do you? How long do you train them, and do you give them certificates?"

"Well, yes, we do train them, but we have no fixed period of training and I think we have given one certificate. This was after about a year's training, and this nurse has gone to Bombay for further training."

No Nurse on Duty.

There was nothing much to learn here, so I promptly drove on to the general hospital. This is a hospital of about 80 beds, and I found a charming staff of what ar called "hospital assistants" ready to take me round and explain to me and to show me everything.

"First of all, gentlemen," I said, "tell me about your nursing."

"We only have one nurse, and she is away."

Here was a big hospital of 80 beds, medical and surgical, male and female, and not a single nurse! How could it be managed? I sat down in the verandah with a little company of white-robed, many-coloured turbaned men standing round me and set to work to cross-examine.

"Who washes the patients in the morning?" I began.

"The patients themselves if they are able, and if not, the ward boy in the male ward and the woman servant in the female ward."

"Who makes the beds?"

"The servant."

"Who removes excreta and sputa?"

"Oh, the bungay comes and does that."

I may explain that in every town there is an outcast class—human pariahs almost—men, women and children, who, being born of the "bungay" caste, must remain of the bungay caste all their lives. No one will touch them. They are not allowed to enter a temple, and they usually have a separate staircase, by which they enter a house to remove the contents of the chambers and of night commodes which take the place of W.C.'s in a land where no sewage system exists.

These are the people who come once or twice a day and carry out from the ward the vessels, covered over with a cloth, and empty them somewhere in the hospital garden. This work is considered so degrading, that no high-caste Hindoo woman who might undertake nursing, would under any consideration do it.

"Well," I went on, "who does the dressings?"

"The hospital assistants, helped by the ward boy."

"How many hospital assistants and ward boys are there?"

"Three H. A.'s, with occasionally two supernumeraries and there is one ward boy to each male ward, and one ayah or female ward servant to each female ward."

"Who does night duty?"

"Ordinarily there is one bungay, one puggy and one ward boy on duty during the night. The other two may sleep, but the duty of the puggy (or watchman) is to make regular rounds of the hospital wards and grounds and outbuildings during the night."

"And if anything is wanted in the wards?"

"One of us is called. If there is any really urgent case in the wards, we take it in turn, of four-hour watches, to sit up in the ward the whole night through."

The Hospital Assistant.

I can quite understand any nurse reading thus far, saying, 'but what is a 'hospital assistant?''

It took me some little time to exactly gauge his position, but I am inclined to put him down now in the category of H. S. and H. P. combined; but to remember at the same time that he bears about the same relation to the lordly, masterful English H. S.—whom every nurse thinks it her privilege and her duty to "take down"—that the "6d. dispensary" doctor bears to the general practitioner. India is a land of cheapness, and, therefore, cheap doctors are as necessary as cheap tailors.

"Hospital assistants" are young men who have received three years' training at certain recognised provincial hospitals in anatomy, and dissecting, and post-mortem, and medicine, and clinical clerking, and dressing, and compounding, and who have passed the prescribed examinations, and have received a recognised diploma of "hospital assistant." This qualifies them for the post of H. S. and H. P. (or resident medical) under a visiting man. A large number set up and practise as "doctors" on this diploma.

### The Ward Boy.

"Who takes temperatures and pulses?" I continued.

"The hospital assistant."

"Who tests urines?"

"If it has to be measured daily the bungay does it in our presence. When any other testing is necessary we do it, but urines are not tested in ordinary routine practice in this hospital."

"Who prepares the theatre?"

"The senior H. A. with the servant, and he also sterilises the instruments."

"Who brings the patient into the theatre?"

"The ward boy."

"And who gives the anæsthetic?"

"The senior H. A."

"What anæsthetic do you use?"

"Chloroform, with a Junker."

"If you had death under chloroform would there be an inquest?"

"No, I do not think so, because the patient voluntarily agrees to its being given!"

"The ward boy seems to me," I said, "to be as important a functionary as any you have in the nursing line. Do you mind telling me your day's work?" I said, on turning to a stalwart man with shaggy black beard and great white turban who was holding my hat, and who answered to the title of "ward boy."

### The Day's Work.

"We come on duty at 6 a.m. We then sweep and clean the wards and make the patients comfortable, make beds and see that the bungays clear everything cleanly away. Go round with the doctor, who comes at 9, and remain in attendance till 11 or 12 or 1, according as there are or are not operations. In the afternoons wait on the H. A.'s and patients and remain always in the ward for whatever is needed up to 8 p.m."

"And meals?"

"We get about an hour off during the day at about 11 or 12."

"How many times do you eat then?"

"Twice a day, at 11 a.m. and 8 p.m."

"And what is your salary for this work?"

"Thirteen shillings and fourpence per month."

"That is in addition to board and residence, I suppose?"

"No; I have a small hut given to me across the garden, but I have to find my own food!"

If there are any Englishmen out of work who would like to apply for the post of ward boy at 3s. 4d. per week and a bare hut found, for 13 hours a day work, they need not expect to get it without considerable competition. I was afraid to ask what the H. A.'s got, but where a nurse-ward-maid man is so ridiculously paid I imagine that the remuneration of an English resident medical would sound colossally munificent.

### A Strange Custom.

In the theatre I found a marble slab let into the wall, and I quote the inscription upon it in order to add that I hope a similar custom will never come into vogue of adorning our operating theatres with memorials of those who die therein. There is no surname and no address to give any clue, but simply

"Phillis."

The tender life was past the reach of art
One night we watched, the next we laid her in the grave.
The patient's sweet submission under pain
Lives as a bright example and relief
To bid afflicted parents smile again
Through memories sweet that hallow all their grief.
Oh let it not be thought that Phillis lived in vain.
Our dear little Phillis born October 31, 1883,
Died in this room on August 27, 1887.

She was so good and sweet in her sickness. She was a pleasant child, and her dear feet were always busy on some kind message. Her nature, like her name, was Love.

### Necessary and Unnecessary Ward Work as Training for Probationers.

By a Late Matron.

A good deal of fragmentary correspondence has lately been published in the columns of the nursing section of The Hospital on the subject of necessary and unnecessary ward work as training for probationers; affording evidence of very conflicting sentiments and standpoints. As a ward sister and matron of some experience I venture to write on what I take to be an important touchstone of enlightened or old-fashioned views of the nursing career, which the title supplies.

### The Power of Matrons.

Actually, though perhaps not admittedly, the framing of rules for the training of nurses is in the hands of matrons of hospitals. "By order of the Board or Committee," no doubt gives the nominal authorship, but such rules and regulations have most probably been drawn up by the matron, and subject to the board's approval, have become law. Therefore, in discussing the abolition or curtailing of ward work as an integral part of a nurse's training, I am addressing myself to a great body of women who in the present or at some future time may be in a position to put their opinions into effect. Tennyson's lines, "The old order changes, giving place to new," do not in spite of the New Woman commend themselves in many ways to our sex. Women love change, but paradoxical as it may sound, are obstinately conservative, and in no department more than nursing—in fact over the régime of some one could appropriately blazon the old words, "As it was in the beginning, is now, and ever shall be," as the keynote of work. Such an attitude of mind, however, needs to be corrected if not entirely relinquished, when discussing nursing problems. The object of education is not the acquisition of facts, but the training of the mind by the knowledge of facts to a habit of intelligent and individual thought, for which the facts form only the foundation of our mental house. So should, in my opinion, a nurse's training lead her, when in authority, not to the reproduction of identical methods by which she learnt—formulated in that too well-known saying, "When I was a probationer I had to do so and so, and you needn't think you're badly used"—but rather to an appreciation of where improvement can be introduced and hardship lessened. The questions to which one would find replies, then, are:—1st. What is ward-work? 2nd. Should ward-work form part of a nurse's training? 3rd. Would it be advisable to have recognised limits in all hospitals as to the description of domestic work required of probationers? 4th. The reasons why ward work forms in many institutions so large a portion of a nurse's duty. 5th. How could the difficulty of the abolition in great measure of ward work for probationers be surmounted?

### What is Ward Work?

In the usual application of the words it means—all those duties, the performance of which is essential to the good environment of patients, but which in themselves form no part of medical treatment or nursing, in strict terms, *i.e.*, sweeping, dusting, polishing, or scrubbing of floors, etc.; just as in cookery, a clean chimney, bright fire, immaculate saucepans and baking tins are necessary to a successful result, yet would not be included in the recipe for a given dish. Having defined what "ward work" is, the question faces us—

Should Ward Work form part of a Nurse's Training?

And I think the reply will be almost unanimous: Yes, it is necessary to the thorough training of a woman in nursing; but push the question further, and ask why it should be required of a nurse, one arrives at quite divergent reasons for the common opinion, and the threadbare and futile answer, "We had to do it," is probably the commonest and most satisfying explanation to the majority. Impossible people, these, whose sympathies run out to the soldier who, being told by the sister when he was lying in a critical condition he was a little better, inquired what day of the week it was. "Sunday morning," said she, to which he answered, "I don't like to 'ear ye say it, Sister, for when me grandmother was very bad, she felt a bit better on Sunday morning, and she be dead on Sunday night." Any change is to such unwelcome and ill-advised. Or someone will say "ward work" teaches a girl how to work, and is good as discipline. An element of truth underlies both these assertions; but one asks, "Cannot these undoubtedly desirable results be attained by means which do not try or exhaust a girl's physical powers and thereby unfit her for nursing duties?" She enters a hospital to acquire proficiency in these, not to qualify as a housemaid, though housemaids are useful in their proper place. I have seen nurses come to the breakfast table after sweeping and polishing floors with heavy scrubbers trembling from sheer exhaustion, and feeling at 8 A.M. that a hard day's work had been accomplished. They "knew how to work," truly. It was my duty as staff nurse at one of the largest provincial hospitals one evening every week to scrub 10 big lockers; the lower half was deep from back to front, and to do the scrubbing efficiently involved getting on one's hands and knees with head and shoulders inside the locker; in that position with cuffs off and sleeves well up I was found by the Medical Officer. I felt a charwoman, not a nurse, and learnt, if nothing else, to sympathise with my fellow creatures who earn their living by charring. This, too, was "discipline," but helped in no way my professional career. To repeat the question, "Why should ward work form part of a nurse's training?" That she may know how to instruct those who are suitable by up-bringing and the accident of birth, for such work. This satisfies common sense, and should be inducement enough to a nurse to acquire during a short time of her probationership a thorough knowledge of a wardmaid's duties, as embraced by "ward work." All who have held authority know that to direct and supervise the work of others is a far stricter discipline than the performance of that work oneself, and the habit of observation which it entails is of infinitely more value than the mechanical doing of certain household duties.

The Third Question.

Would it be advisable to have recognised limits in all hospitals as to the description of domestic work required of probationers? A common standard is much needed. In some hospitals a large part of a probationer's time is taken up with domestic duties—sweeping, scrubbing of floors, lockers, tables, baths, cupboards, washing up of breakfast, tea, or supper crockery, polishing of innumerable brass taps, cleaning of sinks, washing of gas globes, etc. In another institution some only of these tasks are expected of a nurse. It would be a distinct step in advance if matrons were to agree that the majority of these duties should not be alloted to the probationers, exception perhaps to be made in some particulars for the operating theatre; a multitude would yet remain that have close connection with nursing, *e.g.*, brushing and carbolising of beds, cleaning of lotion bowls, instruments, many and various, dusting, etc.

House Work.

Why does ward work occupy so important a position in a nurse's training? Two main factors account for it—the committee's desire to keep down expenses and the spirit of emulation among sisters of different wards. Having a given number of women to train and a large amount of women's work to compass, and bearing in mind that the class of work is familiar to many of the women, it would seem not unnatural to fit the horse to the cart and arrange that the women should undertake the work. Natural and reasonable but for one important particular—the women came to be trained as nurses, and the work which is demanded of them for a large portion of their probation is housework pure and simple. The interests of the institution override the just claims of the probationer. There is no force in the argument that a hospital is a charity, and must be worked on the most economical lines, and all unnecessary expenses cut down. Probationers are admitted to hospital, not as assistants in a philanthropic work, but as students of a profession, as emphatically as a young man is articled to a solicitor; and it would be as logical to expect the latter to sweep the office and clean the windows as it is to compel nurses to wash up tea-things and gas globes. Long custom is a difficult enemy to meet and vanquish, but if the standard of nursing is to be maintained in an upward direction, menial domestic work must be relegated to wardmaids or their equivalents.

The Rivalry of Sisters.

Secondly, the rivalry between sisters of different wards. This leads to a ceaseless striving after polishing and cleaning *per se*. No advantage accrues, to the patients or probationer, but the sister has the satisfaction that her brasses and furniture are the best polished in the hospital. Not at all a blameworthy ambition some would say; but a false one nevertheless. Far better to sacrifice a little self-congratulation on such a point, and divert a probationer's energy to more distinctly nursing duties.

Practical Difficulties.

How could the difficulty of the abolition in great measure of ward work for probationers be surmounted? By the employment of a few extra charwomen which would relieve the wardmaids of some work, setting them free to take over duties now apportioned to nurses. Additional expense would be incurred, but the outlay would be more than compensated by a higher standard of health in the nursing staff; greater professional skill, because of more leisure to appreciate the real work of nursing; less grumbling, because of diminished cause of complaint, and last, but not least, more sympathy between nurse and patient, the latter not feeling so keenly "Nurse is so busy, I can't bother her;" and the nurse conscious that a few words of chat with her sick charges, does not mean a reproof for taps not cleaned to time, or ink-pots or lamps below regulation standard of brightness. How many probationers can corroborate the sad remark: "There was such a lot of cleaning to do, there was no time to attend to the patients." Transition is not revolution, and many suggested changes could be accomplished without friction or heart-burning—an open mind can grasp and achieve much. One other aspect of the subject remains untouched. The breakdown of many girls on first going to hospital, is due more often than not to "ward work." Is not good potential nursing material, perhaps of the best kind, thus lost to the profession? A yacht made into a coal-carrier is a parallel example of the inappropriateness of work often seen in a hospital.

### A Souvenir of Marlborough House.

THE nurses of the Royal National Pension Fund have just received the intimation that the proprietors of THE HOSPITAL have prepared a beautiful and most interesting souvenir of the now historical receptions at Marlborough House where Queen Alexandra has presented certificates to so many of her nurses. In view of the fact that the reception last July must necessarily be the last over which Her Majesty would preside at Marlborough House, and in response to the wish often expressed by members of the Pension Fund to possess a souvenir the proprietors took steps to procure a series of photographs of all the striking scenes before and after the ceremony, and also to have a special drawing made of the function itself. The photographs have been reproduced in a highly artistic manner, mounted suitably for framing, and form part of a handsome album, in which is also included fine portraits of King Edward and Queen Alexandra, of Lady Rothschild, President of the Junius S. Morgan Benevolent Fund, and of the chairman, the founder, and the secretary of the Royal National Pension Fund for Nurses. The letterpress desoribes in detail the various delightful gatherings at Marlborough House, and tells the story of the Fund itself. The ceremony in July is depicted with amazing accuracy and ability in a drawing from life by Mr. Maurice Greiffenhargen, who has made remarkably good likenesses of many of those who took part in it. They will be easily recognised by all who know them. This drawing has been reproduced as a facsimile, and the execution of the picture is so faithful that the artist declares it to be the best reproduction he has seen of any of his works. No pains have been spared by the proprietors of THE HOSPITAL to obtain for the members of the Pension Fund all the features that could enhance the value of a permanent souvenir of this Fund and of the charming fêtes at Marlborough House. In their efforts they have received the hearty and valuable co-operation of the officials of the Fund. The portrait of Mr. Dick at work in his office, specially taken for the occasion, will be a particularly acceptable feature to the nurses, to whom he has always been a kind and

THE QUEEN PRESENTING CERTIFICATES TO THE NURSES.

courteous friend. Of the small illustrations accompanying this announcement, one shows the nurses and other guests at tea in the grounds at Marlborough House, and the other is a miniature reproduction of the large plate representing the ceremony of the presentation of certificates. The album and the plate are being issued by subscription to the nurses of the Pension Fund, and their requirements will first be met, as only a limited number of either the album or the plate are available. But if any of our readers who have not had an opportunity of subscribing, express a wish in writing to the manager to receive a copy of either, or both, the

AT TEA IN THE GARDENS AT MARLBOROUGH HOUSE.

request will, if possible, be complied with. The price, post free and carefully packed, of the album is 5s., and of the plate 10s. Applications for copies should not be too long delayed, as the productions are too expensive to reprint.

### Presentations.

BRISTOL GENERAL HOSPITAL.—Miss Lewis (Sister Riddle), who was trained at the Bristol General Hospital, after 17 years' service, has given up her work through ill health to take a long rest. Miss Lewis was sister in the casualty and O. P. D. for some years, and for the past nine has been sister of the male surgical wards. On leaving the hospital on November 1st she was presented with £40 from the committee for long and loyal service and from the nursing staff, the officers past and present, a clock and purse containing £10 10s. She will be much missed by all; the patients have lost a good and sincere friend and the committee a valuable sister.

MERCER'S HOSPITAL, DUBLIN.—Miss Margaret M. O'Flynn, on leaving the Mercer's Hospital, Dublin, was presented with a beautiful dressing-case by the Resident Medical Staff.

STRATFORD-ON-AVON HOSPITAL. — On Wednesday, the 30th ult., Miss P. J. Brady was presented with an afternoon tea-set with tray, and a travelling clock, on the occasion of her appointment as nurse-matron to the Pembroke Infirmary. The presentation was made on behalf of the honorary and nursing staff of the hospital and a few friends. Miss Brady had almost completed nine years as staff nurse at the Stratford-on-Avon Hospital, and she carries with her the cordial good wishes of all with whom she has worked.

THE "REST," PORTHCAWL.—Miss E. Gray, the lady superintendent of the "Rest" Convalescent Home, Porthcawl, Glamorganshire, who is leaving the institution to which she has been attached since March 1900, has been the recipient of a number of presents. The patients have given her an illuminated address, Miss Sansum and the nurse a silver pencil-case, the servants an inscribed Bible, and the female patients a silver bread-knife and platter. The various presentations were made to Miss Gray at a meeting last week, not the least interesting feature of which was her own address of thanks to all who had shown their appreciation of her services.

### A Book and its Story.

### ONE OF THE QUEEN'S MARIES.*

Among the many recently published books dealing with life and times of the ever-interesting Mary Queen of Scots, none, perhaps, is more incidentally charming than the love romance of Mary Hamilton, who became one of the Queen's Maids of Honour after her return to Holyrood upon the death of her French consort, Francis II. The tragically pathetic narrative of the speedy wooing and hasty marriage of the thoughtless, lighthearted Mary Hamilton, and all that it meant to her in the future, is told in the quaintly simple language of the time by "her very dear comrade, friend, and kinswoman Anne Cunninghame," who was appointed her waiting-woman and companion at the age of sixteen. Mistress Anne was a keen observer, and she had a ready tongue, and a quick sense of humour, as her narrative, written sometimes in the first person, and occasionally in the third, shows. She proved herself an ideal *confidante*, and the author displays much skill in his delineation of her bearing, in the very difficult part which fell to her, when sharing the fortunes of her beloved mistress. Around the birth of Mary Hamilton a mystery hangs that is not removed until the end of her history. In the opening chapter she is the subject of a conversation between the old laird, Patrick Hamilton, and James, Earl of Arran. "Patrick Hamilton and his dame were childless, and gliding swiftly down the steep slope of life's later years; and so it came about that the Earl of Arran, knowing the loneliness of their state, and the pure lealty of their hearts, thought that they and the little Mary would be a plain comfort one to the other, and so made mention of the child to the old folk, feeling, as it were, their humour in the matter." A winning little maid he pictured her, and one that would bring sunshine into the life of any house. "This little maid of yours that you speak of, how old is she?"

"Four years come Lammas," answered my lord, "and already more winsome of speech and manner than bare words can paint. None that have eyes can see her without instant love." To the reception of the little maid the old people demurred, for the grey old house, whose walls had never been brightened by the sunny smiles and happy voices of children in their earlier years, was no fitting place they felt to bring a child to now that they themselves, were outwardly, as grey and dour as the house itself. But the Earl knew full well the kindly hearts that beat behind the grim exterior, so he pressed his cause regardless of objections and protests, through which gleamed the longing to take the child. "This is a dull, joyless kind of house," said the good laird, rising from his seat to throw more logs upon the fire. "Aye, and dull, grey, joyless old bodies they are that live in it," said the dame, plying her needles with great zeal. . . . "It's ill for the young to be housed with those whose backs have long been turned to joy and laughter."

"Not so," said my Lord Arran, rising and facing the matter now with a straight front. "It is better for a child to be housed with good, honest, kind souls than to be cast away at the outset into the rough byways of the world. Take the child, kinsman, and she will bring God's blessing on you, and you on her."

And so it came about some ten days later "My lord rode up with the little maiden on his saddle-bow." After confiding her to the keeping of her new friends he rode away. No one knew with certainty who were the parents of the little thing. The Earl of Arran had brought her from France. He was there at the time of the great gale in which her parents were supposed to have gone down. The little maid was all alone in the world, and after arranging their affairs he brought her back with him. "Until the day that Arran arrived in Edinburgh with the child beside him in a horse-litter, none had ever heard that issue had been born to John Hamilton of Blair and his wife; but, seeing the child, men thought no more of the matter beyond wondering at their own lack of knowledge and at the silence of the Hamiltons of Blair." At the age of twelve, when the old laird has persuaded his kinswoman, Dame Cunninghame, to part with her daughter for a handsome consideration, we have the first glimpse of her in a little pen portrait given by her observant companion Anne. "She was tall for a woman, though not within an inch of my own great height, and as straight and upright as a ship's mast. She walked with quick short steps, holding herself a little stiffly, but with grace beyond words. Her face was wonderfully round till it reached the chin, and there it ran quite quickly into a sharp point that essayed to turn upwards, and a perfect fellow to the chin was the nose above, very sharp at the point, and also inclined ever so little skywards. For the rest her face was very pale; she had grey eyes and a wonderful wealth of dark brown hair, and brows that were straight and thick. . . . She was at all times very grave and staid, but with an amazing skill in mummery, and would ape the tricks and moods of any who sought entertainment at Andrew's Knowes, . . . and when we were free of company entertain me till the tears coursed down my cheeks from laughter and I was fain to pray her cease, for love of mercy, before my sides were clean cracked." Had the demure little Mary Hamilton wedded James Hamilton, their nephew, the suitor selected by her adopted parents, her life would have been other than it became, and her story as written by her kinsman of another generation would have been untold. But it is plain to see that this suitor was not to Mary's mind at all. "James, poor, simple, stiff-necked James, knew nothing of the way in which his lady-love used him behind his back; not that there was any malice in it. Malice and Mary Hamilton had at no time any part in common, but when a maid makes laughter out of the man that seeks her hand, he had best go elsewhere, as the world has found out a long day since"; a conclusion in which Mistress Anne was correct enough, and one that holds as good to-day as then. But poor little Mary, with her staid manner and piquante face, rejecting the suitor at home goes forth from sheer ennui, in company with her youthful duenna, to fall upon the most luckless fate that she could have encountered. A retired life, lived in sweet seclusion, warm feelings, a love of harmless masquerade, that never entirely left her in the grievous days that came later, all helped on the fate that she and the youthful lover "Henry Stuart," Lord Darnley, were preparing for themselves. The fidelity and loyalty of her faithful Anne Cunninghame "the dearest friend I have and whom I love with all my heart," is a touching study of a many-sided character, true to the death of the mistress who loved her so well. The whole tragedy and pathos of the romance centres around the secret marriage which Darnley is represented as having persuaded Mary Hamilton into, previous to his having become king, as husband of Mary Queen of Scots.

The story of Mary Hamilton is inimitably told, and the reader's interest is kept alive throughout. Everyone who appreciates historical romance should read Lord Ernest Hamilton's attractive study of Mary Queen of Scots' maid of honour and rival.

* "Mary Hamilton." By Lord Ernest Hamilton. (Publishers: Methuen and Co. 1 vol. 6s.)

### Echoes from the Outside World.

### AN OPEN LETTER TO A HOSPITAL NURSE.

Only royal personages confer, as well as receive, honours upon their natal day. The most notable of those bestowed by the King upon the ninth of November was the title of Prince of Wales which he conferred upon his only son. Most of us who knew little about the laws of the constitution of our country at the time of Queen Victoria's death imagined that the Duke of York would be called Prince of Wales directly his father became King, but we found to our surprise that the title was not hereditary, but had to be made the subject of a fresh grant every time that a new monarch came to the throne. In our childhood's days we all learnt the supposed origin of the name, how Edward I. presented his newly-born son to the Welsh chieftains at Carnarvon Castle in fulfilment of his promise that he would give them a prince "free from any blemish upon his honour and unable to speak a word of English;" but alas! like many another of the pretty stories of the past, it is now said to be apocryphal. Several English Kings, notably, Edward III., Henry VI., and Edward VI., never bore the title of Prince of Wales, while there is much evidence that Queen Mary was Princess of Wales before her accession. Two of the most striking points are that Linacre dedicated his "Rudiments of Grammar," published in 1523, to Mary as "Princess of Cornwall and Wales," and that an inscription in the chapel at Ludlow speaks of a bishop as "President in the tyme of the Ladye Mary, Princess of Wales." This is the only instance of a woman bearing the title. I also notice that amongst the long list of birthday honours conferred by the King, which includes a baronetcy upon the late Lord Mayor (Mr. Alderman Frank Green), there is only one lady, Mrs. E. Y. Firth, of Bombay, who is given the Kaisar-i-hind medal for public service in India.

---

As usual, Lord Mayor's Day began with a Show and ended with a dinner—one pleasure for the many and the other for the few. The Show was witnessed by more than the ordinary number of juveniles, the weather being exceptionally fine and open. A revival warmly welcomed was the inclusion of men in chained armour riding on horse-back. Apparently the pageant in the morning afforded more general satisfaction than the banquet at the Guildhall in the evening. I do not mean that the guests of the new Lord Mayor did not appreciate their dinner; but the speech of the Prime Minister, which is supposed to be the chief attraction on the occasion, has provoked a lot of grumbling. Yet he seemed to me to take a very sensible view of the position in South Africa, and to give all the guarantees in his power to bring the war to a termination as soon as possible. It is amusing to note that because Lord Salisbury did not fulfil the prediction that he would adopt the language of a pessimist, and spoke in a cheerful tone, he is accused of unjustifiable optimism.

---

The appointment of Canon Gore as Bishop of Worcester is an event of great interest, and has given rise to widespread criticism, chiefly, but not altogether, friendly. A singularly powerful preacher, a man of remarkable force of character, and a leader of religious thought, it is quite in accordance with the fitness of things that he should be elevated, in the prime of life, to the episcopal bench. Decidedly he is the most popular of the Canons of Westminster, and he will be much missed by people who are in the habit of worshipping at the Abbey. It may also be assumed that he will speedily make his mark in the Midlands. He is not unacquainted with Birmingham, where he was the guest of that distinguished Nonconformist the late Dr. R. W. Dale, and although the theological views of Mr. Chamberlain and Dr. Gore are supposed to be wide as the poles asunder, it is said that the Colonial Secretary is extremely pleased with his nomination. High churchman as the new Bishop is, no one has ever accused him of being narrow-minded, and he may be relied upon to rule his diocese with kindly impartiality.

---

A successful Bazaar was held at the Empress Rooms on Friday and Saturday last in aid of the Building Fund of the Gordon Hospital, Vauxhall Bridge Road. There were a great number of stalls, one of which was called the Hospital Stall. It was presided over by Miss Maxwell, the matron, and did particularly well as most of the things were very moderate in price. The lucky bag was under the charge of Nurse James. The provision stall was so much sought after that at the end of the second day the stock was literally sold out. The Serenaders, who are much sought after just now, gave three performances each day in aid of the funds, all of which were much appreciated. The troupe consists of two ladies and three men, all masked and attired in strange garments of red and black and gold. The Ladies' Amateur Harp, Mandoline, and Guitar Band also played during the afternoon and evening, and the unaggressive character of their music was especially suitable to the occasion, as it aided conversation and the important business of buying and selling instead of proving somewhat overpowering, as a band of wind instruments is apt to do.

---

Kate Greenaway's name is such a household word, even amongst those who know little of artistic circles, that her death naturally finds an echo in our record of events. She has long lived at Hampstead in a very pretty house designed and built for her by Norman Shaw, which was always proudly pointed out to strangers by the local inhabitants anxious to draw attention to the fact that a large number of celebrities reside in that northern suburb of London. Up to the end her pencil was busy. As lately as last year, in conjunction with the authors of "Elizabeth and her Garden," she produced a volume for children entitled "The April Baby's Book of Tunes." Perhaps her best-known work, or at least the one which first brought her into notoriety was "Under the Window," and that was quickly followed by "Kate Greenaway's Birthday Book." Her illustrated almanacs were also much thought of, but it was as a revolutioniser of children's dress that she achieved perhaps most fame. Her dainty little boys and girls, arrayed in the costumes worn by the "grown-ups" in 1800, aroused so much admiration that the designs were at once extensively copied, the more so when it was found that even plain children were rendered presentable by this artistic mode of treatment. It has been said of her that she "dressed the children of two continents" and the French nation especially were enthusiastic about her methods.

---

A few weeks ago I was dwelling on the fashionable "plate" hats. They are as much worn as ever in velvet and Oriental satin, often with guipure lace let in round the crown to make them more dainty; but of course there are some purchasers who do not care for the particular shape—they look best on tall people—and for their sakes two or three different ones are also introduced. One of the most remarkable is a small round turban hat something like the "Clara Butt," formed entirely of feather rouleaux wound round and round. Pheasants feathers or the wings of darker birds are utilised for this purpose, and one particular hat of dark greeny-black feathers was coiled so tightly as to be extremely suggestive of a peep at the Zoo, where the snakes often lie twisted round and round in much the same manner. Perhaps when a bow of bright ribbon is placed on the pile, the striking resemblance to a sleeping serpent might disappear. Naturally, however, little trimming is required. The flat feathers are most desirable for November fogs, as there is no possibility of their coming out of curl. Soft felts with long hair are much worn as also are fur hats, and some are most remarkable in shape. The "tricorne" or three-cornered are very pretty on a curly or frizzy head of hair but do not suit smooth locks. Many felt and cloth hats are tied up and laced up at no end of queer angles, but these require a pretty face beneath not to look extraordinary rather than becoming. The great fault of many of the hats this season is their weight, of which those whose hair is not too luxuriant should beware. Nothing except downright illness conduces to the serious falling out of hair so much as too heavy headgear.

### Everybody's Opinion.

[Correspondence on all subjects is invited, but we cannot in any way be responsible for the opinions expressed by our correspondents. No communication can be entertained if the name and address of the correspondent are not given, as a guarantee of good faith but not necessarily for publication. All correspondents should write on one side of the paper only.]

### ISOLATION HOSPITAL, FARNHAM.

"Miss E. Berry" writes that she was nurse in charge of the Willesboro' Sanatorium, East Ashford, Kent.

### THE TROUBLES AT CROYDON INFIRMARY.

"Anxious" writes: Seeing that the affairs of Croydon Union Infirmary are still far from being satisfactory, I should like to know who would sign a nurse's certificate at the end of her three years' training, as the matron is, I presume, suspended from her position as superintendent of nurses. Will you kindly tell me whether the Local Government Board would recognise a certificate without the matron's signature? If not, is it advisable for anyone to enter there for training?

### A WARNING.

"Nurse C." writes: On Monday evening, November 4th, on arriving in London from abroad, I went to a certain hotel in the West Central District. The hotel being full, the secretary kindly gave me the address of a boarding house, and said I might leave my trunk at the hostel until the morning. When I called for it next day I found to my great dismay that the lad who had carried my bag had returned to the hostel that night, told them I had sent him for my trunk, and that the servant had most foolishly given it to him. I have put the matter into the hands of the police, but as it was a foggy night I could not see the lad very distinctly, and I am afraid I am not likely to see my trunk again. Unfortunately, it contained my most valuable possessions, some of which can never be replaced.

### THE NURSING OF OPHTHALMIA NEONATORUM.

"A. M. H." writes: In reply to "H. H.'s" letter of November 2nd on ophthalmia, I fail to see what help it is beyond telling in what position to hold the child, and I think it might do immense harm if it fell into the hands of an ignorant person or even a careless nurse. I am a certificated monthly nurse, and we were always taught to thoroughly cleanse out babies' eyes (keeping the bad eye lowest) with boracic solution every half hour if necessary, nitrate of silver being used by the surgeons or sisters only. Since then I have cured two or three cases of ophthalmia in France, as it is very prevalent here, and often quite an epidemic, with boracic only, by doing it frequently, of course using all antiseptic precautions. It may be well for a certificated ophthalmic nurse to use nitrate of silver, but is it wise to recommend it, even with the proviso "that the nurse will be told by the surgeon"?

### NEGLECTED NIGHT NURSES.

"A Night Nurse" writes: Yes, "Sympathiser" is quite right when she speaks of the poor night nurses being forgotten. What is the reason of the neglect? The matron has been on night duty herself; is it so long ago that she forgets what she had to go through, when in training? It is frequently the lot of a night nurse to be called for something as soon as she goes on duty, and perhaps to be kept busy all night, accidents coming in, fomentations and other dressings to do. Besides all this, I know that some night nurses have to clean all the brass in the ward and do whatever stock is put out for them. Surely after a hard night's work they might have a good meal and a fire in their rooms to warm them; many a poor nurse cannot sleep for the cold. Then they generally have to go without food from 8 a.m. till 8 p.m., unless, as "Sympathiser" says, they buy something tasty: and some are not in the position to be always spending money. As regards getting nights off, some think themselves lucky to get one night in three months. Then, if a nurse knocks up or looks bad, she is told she is not strong enough to continue nursing.

### THE NURSE'S NEVER.

"Brighouse" writes: "The Nurse's Never" is, to say the least of it, intensely amusing, and quite enough to lead one to think the writer of it knows precious little of nurses or nursing. Who in the world would ever dream of allowing patients to take their own temperature, or what nurse would put a hot-water bottle in a bed minus its flannel bag, or leave food by a patient's bedside, and who but an idiot would put strong carbolic acid in a bed-pan previous to giving it to a patient? Surely it would be more applicable to name the paper "The Fool's Never," or "The Stupid's Never," and leave nurses out of the question. Everyone knows that common sense is one of the chief essentials which make a nurse. Consequently no hospital nurse, or even private nurse, could be guilty of such things as are named down the columns, as it would be quite against her nursing instinct, unless it be a few of the "balmy" type who somehow have managed to scrape through a training. May be, the advice given is meant for that class of nurse. But why not call it "The Balmy's Never."

### ARMY NURSING IN SOUTH AFRICA.

"An Army Reserve Sister" writes from Burghersdorp Cape Colony: After not seeing The Hospital for some months I felt like meeting an old friend when I picked up a copy lying about belonging to one of the other sisters at my fresh station. I have been reading the number for September 28th, and feel moved to write to you about Army Nursing and Army Nursing Sisters. I most strongly object to Lady Dudley or anyone else appealing for funds for Reserve Sisters. We are paid most liberally, and with little effort can save quite enough to take a holiday on our return. Some may land without cash, owing to deferred pay, but a loan would suit them best. It is kind to send the sick to a nursing home and to provide suitable rooms for strangers, but money help is not needed. At both Port Elizabeth and Wynberg the warm underclothing sent out for the Nursing Sisters was given to the English refugees, though letters of thanks were sent not to hurt the feelings of the donors. If sisters up country were glad of them (being shopless) they were well able to pay, and if these good ladies could see the "carryings on" of many nursing sisters, they would not be so eager to help them. I have not seen anything actually wrong, but very much that is unseemly. Indeed, I wondered whether we were sent out to nurse the men or amuse the officers. Many young ladies can do both equally well, and it is in the slack times that most nonsense goes on. But you would be astonished at the careless way work is done, and to see the many duties relegated to the much-abused orderly who does it with a sneer, for he and his soldier patient, are severe judges, over-quick to think and speak evil. You may not have noticed that with the large increase in nursing sisters there has been no alteration in their duties, which still remain those of superintending a large number of patients, so that anything more in detail depends on each sister's conscience and ideas, which differ. Of two irregular sisters one told me that she never thought of washing a man's face, that was orderlies' work; the other made a practice of sponging down her bad patients in the evening as conducive to a night's rest, and "not to hurt the orderly's feelings," he gives them a "dab" wash in the morning. In a military hospital so many things are said to be "never done in the service" that it seems as if the less one does the better. I may add that appliances which were old-fashioned 10 years ago are still in use, that some are quite unknown, and that critical cases are left to themselves for an hour three times a day; "it is always done in the service," is the all-sufficient explanation.

### THE SUPERINTENDENT NURSE AT THE SKIPTON UNION INFIRMARY.

"T. Gavan-Duffy," of Skipton, writes: A journal which has served the nursing profession so long and so well as The

Hospital will, I am sure, allow me to put the facts of the above case before your readers, and thus help in rescuing the reputation of this lady from the charges laid against it. At the fortnightly meeting of the Skipton Board of Guardians held on Saturday, October 12th, Lieut.-Colonel Maude made a charge against the superintendent nurse which in effect was that (1) she did not report to the Master of the House the serious condition of her patient; (2) that although having received a stamped envelope from her patient's husband, she never answered his inquiry as to his wife's health. Strange to say, this statement was accepted and the superintendent nurse condemned without a word of inquiry or investigation on the part of anyone! The nurse was not questioned; she was not present; no intimation whatever of the charges had been made to her. The statements were made, accepted without inquiry, and flashed all over the country. The moment inquiry was made every one of the charges dropped to pieces like a house of cards. The superintendent nurse gave an indignant and well-substantiated denial to every one of the allegations. The charges were, however, formally brought before the House Committee, who after investigation reported that "they had come to the conclusion that no intentional wrong had been committed by anybody"; but this decision was too ambiguous to satisfy anyone. It certainly did not satisfy the nurse, for against her a great "wrong" had been done, whether it was "intentional" or otherwise. In your note you say the patient "had been dead several days" before her husband learned of his loss. But the following chronological record of the case will remove that and many other errors, and demonstrate very clearly that the superintendent nurse efficiently discharged her duty in every detail:—

Saturday, Aug. 31st.—Patient admitted "in a very weak condition."

Sunday, Sept. 1st.—A written report stating that the patient was "very weak and very ill" was handed to the Master.

Wednesday, Sept. 4th.—Extract from report book: "Patient very poorly, very weak. Written report for second time handed to the Master."

Saturday, Sept. 7th.—Patient's husband wanted to visit her at infirmary, but persuaded by Col. Maude not to do so, "as she had only been in the infirmary a week."

Monday, Sept. 9th.—Letter addressed to patient received at infirmary signed "Wilcock," which was not her husband's name. This letter was opened by an assistant nurse, who read its contents to patient, being then told to put it in the box, as "the old man would be coming on Saturday."

Monday, Sept. 9th.—Extract from report book: Patient "very ill."

Thursday, Sept. 12th.—Extract from report book: Patient "much worse."

Friday, Sept. 13th.—Extract from report book: Patient "very much worse."

Saturday, Sept. 14th.—Patient died about 1 A.M. At 6.30 A.M. the death was reported to the Master, whose duty it undoubtedly was to communicate to the deceased's friends. At 2.15 P.M. on this eventful Saturday, superintendent nurse Neil was going out when she met the deceased woman's husband (whom she knew), and he said he was going up to have a look at his old woman. The nurse, never dreaming that he did not know of his wife's death, thought he was going to the mortuary until he startled her by asking, "How was the old woman?" Then the nurse said, "Good gracious! have you not had a letter or telegram?" He replied, "No; what has happened?" And the nurse replied that she was "sorry to say his wife died that morning, and that a letter or telegram must be waiting for him at home;" but there was neither for him. Although this death was duly reported at 6.30 A.M., no intimation whatever was made to the husband of the deceased. Yet no one can contend that, having made her report, it was further the nurse's duty to write or wire to the deceased's friends. The facts, then, show that the report book, to which the Master had full and free access, was properly kept by the superintendent nurse. On two separate occasions written reports, detailing the condition of the patient, were handed to the Master, who took no action regarding them; no letter was received at the infirmary, written or signed by the patient's husband; no letter of any sort or kind connected with that case was addressed to the superintendent nurse, who in every particular did her duty, and did it well. Nurse Neil is no evanescent meteor in the nursing profession. Step by step she has won her way up to the top by skill, diligence, and perseverance. After a thorough training in a Poor-law infirmary she spent three years as district nurse, bringing hope and mercy and succour to the poorest of the poor in the slums of Manchester, changing from that toilsome pursuit to private cases, and from that back again to Poor-law infirmaries, in which she has spent the past six years, winning deserved approbation, esteem, and promotion in each appointment. During the three years she has occupied her present position she has endeared herself to every patient who has come under her charge, and when it is added that a special committee of the Skipton Guardians have now completely exonerated Nurse Neil from all blame in connection with this case, it will be admitted that she and her friends have cause for indignation at the cruel treatment she has been made the victim of.

### Wants and Workers.

Infants' Clothing.—Will any kindly disposed ladies having no further use for infants' or little children's clothing, no matter how old, allow District Nurse, Cawston Lodge, Haverland Park, Norwich, to have them for free distribution among the poorest of those among whom she works? There are so few to help and the need is great. District Nurse thanks the kind friend who sent parcel of boot and shoes, etc. (name and address unknown). They were most acceptable.

Wicker Spinal Carriage.—Edith P. Wood, District Nurse, 3 Winchester Street, Sherwood, Notts., writes:—"A family in my district had, at considerable cost, and some privation, purchased for the use of their mother (rendered utterly helpless by rheumatism), a wicker spinal carriage. This has been used just six times, and now, unfortunately, there is no further need for it. It is a light running rubber tyred carriage, 6 feet by 27 inches, and the family are anxious to sell it so that they may realise at least a little of the money they could so barely afford."

### Death in Our Ranks.

The death of Miss Mabel Florence Thomas, at the early age of 25, is announced. Miss Thomas was trained at the Kensington Infirmary, and had held the appointment of Sister at the same institution for over a year. Leaving last June she spent a holiday at home in South Wales, prior to taking up fresh duties at the Seafield Nursing Institute, Cardiff. While engaged on a case she was taken ill and returned to the Institute, where it was found she was suffering from "abscess of the lung." An operation was advised but she was too ill to take the risk, and after five days' terrible suffering she passed to her rest on Saturday, November 2nd, death being due to "septic pneumonia resulting on the bursting of the abscess." The sad news of her premature death was received with great sorrow among those with whom she had worked so long.

### Where to Go.

Bazaar at Torquay, November 27, for the benefit of Rose Hill Hospital for Children, Babbacombe. A pen-and-ink sketch of a proposed new building, which is estimated to cost £2,800, will be exhibited.

Camden Theatre, Camden Town.—Grand Matinee in aid of the Building Fund of the Hampstead Hospital, Tuesday, November 26th, 2 P.M.

### Appointments.

Bristol General Hospital.—Miss A. M. Sampson has been appointed sister of Riddle ward, and Miss A. Curry, sister of the children's ward. Both were trained at the Bristol General Hospital.

Craigleith Parochial Hospital, Edinburgh.—Miss M. G. Watkins and Miss Mary Annette Secretan have been appointed charge nurses. Miss Watkins was trained at Mill Road, Liverpool, for three years, where she was afterwards charge nurse. She has also done private nursing in Newcastle-on-Tyne, and been charge nurse at Barnard Castle Fever Hospital, and at Bishop Auckland Fever Hospital. Miss Secretan was trained at Bolton Infirmary for three years, and has since been charge nurse at Noble's Hospital, Isle of Man. She has also done private nursing at Newcastle-on-Tyne and Penrith.

Ipswich Union Infirmary.—Miss Janet D. Everett has been appointed nurse. She was trained for two years at the Cancer Hospital, Brompton, and for three years at Southwark Infirmary.

Manchester Royal Eye Hospital. — Miss Marjory Sutherland has been appointed matron. She was trained at Newport and Monmouth Hospital, and has since been sister at Manchester Royal Eye Hospital, and assistant matron of the Birmingham and Midland Counties Training Institution for Nurses.

Moreton-in-Marsh Cottage Hospital. — Miss Amy Gough has been appointed nurse-matron. She was trained at the Bristol Royal Infirmary for three years. She has since been charge nurse at Park Hospital, Lewisham, and the General Hospital, Birmingham, and sister and assistant-matron at the Eye Hospital, Bristol.

Royal Hospital for Sick Children and Women, Bristol.—Miss Margaret M. O'Flynn has been appointed surgical and theatre sister. She was trained at Drumcondra Hospital, Dublin, where she remained a year and a half as charge nurse. She also held the post of sister of the female surgical and gynæcological wards at Mercer's Hospital, Dublin, for two and a half years.

Royal United Hospital, Bath.—Miss Florence Barker has been appointed nurse on the private staff. She was trained at the Poplar and Stepney Sick Asylum, where she has also held the post of staff nurse.

St. Pancras Workhouse.—Miss Elizabeth Tudor Hogg has been appointed assistant nurse. She was trained by the Meath Workhouse Nursing Association at the Crumpsall Infirmary, Manchester.

Stratford-upon-Avon Joint Infectious Hospital.—Miss L. E. Freeman has been appointed matron. She was trained at Shrewsbury Infirmary and at the Isolation Hospital attached to Chester Infirmary. She has since been matron of the Borough Hospital, Kidderminster, and matron of the Belper Joint District Hospital.

South-Western Fever Hospital, Stockwell.—Miss Lottie Bradley has been appointed charge nurse. She was trained at the Poplar and Stepney Sick Asylum.

### To Nurses.

We invite contributions from any of our readers, and shall be glad to pay for "Notes on News from the Nursing World," or for articles describing nursing experiences, or dealing with any nursing question from an original point of view. The minimum payment for contributions is 5s., but we welcome interesting contributions of a column, or a page, in length. It may be added that notices of entertainments, presentations, and deaths are not paid for, but, of course, we are always glad to receive them. All rejected manuscripts are returned in due course, and all payments for manuscripts used are made as early as possible after the beginning of each quarter.

### For Reading to the Sick.

### COMPASSED ABOUT WITH A GREAT CLOUD OF WITNESSES.

They parted here in weakness, and suffering, and gloom
They meet amid the freshness of Heaven's immortal bloom:
Henceforth in ever-during bliss to wander, hand in hand,
Beside the living waters of the still and sinless land.

Oh! who can tell the rapture of those to whom 'tis given
Thus to renew the bonds of earth, amid the bliss of Heaven?
Thrice blessed be His holy Name, Who, for our fallen race,
Hath purchased, by His bitter pains, such plenitude of grace.

*Rev. W. Alexander, D.D.*

We are part of a great "Family." Our elder brothers and sisters are out of sight; a thin veil hides them from us. But they are close to us; they are round about us, as a "cloud of witnesses." They see us, and know us, and love us. Probably, they mingle their intercessions with ours. They are allowed to minister to us, it may be, under the Angels. But certainly, on the word of God Himself, they are one with us, and near to us. They are, even as *we* are, "in Christ;" branches in the One Vine, members of the One Body only, they have been set free from certain difficulties by which *we* are hindered.

Many altogether fail, because their inward gaze is fixed on the clouds which perpetually hang around the horizon of their life.—*Bishop Wilkinson.*

"Unseen, yet not unfelt, the chain,
The Golden Chain encircling every breast,
Which, ne'er to meet on earth again,
Saints that are gone, and that remain,
Knits through all ages in communion blest."

*Lyra Sanctorum.*

Yet the true life is beyond. Beyond is the only enduring vision. Beyond lies the eternal. Beyond is the interpretation of the mystery of the dispensations, the secret of the all-ruling Mind, the only clue to what seems the ravelled skein, but is really the marvellous interlacing of the manifold lines of the Providence of God. Beyond is the true "Light" which is the "Life of men," which from behind the cloud shines through and within the soul that looks for It—the Light which irradiates the emancipated spirit's secret recesses, kindles its deeper thoughts, and sheds a halo around all the circumstances, even the most trying passages, of this passing state. Beyond is the full fruition of that of which all present hope is the feeble reflection. Beyond is the Joy, on the threshold of which the believing spirit waits, which thrills even now through all its inner senses, growing ever more and more into the delighted consciousness of "the powers of the world to come." And in this anticipated Joy sorrow loses all its desolateness, pain ceases to be overpowering, loneliness is cheered, life's burden becomes easy, the bitterness of death is past.

Thus encompassed with the "great cloud of witnesses, let us look unto Jesus, the author and finisher of our faith; Who, for the joy which was set before Him, endured the Cross, and is set down at the Right Hand of the Throne of God."—*T. T. Carter.*

### Notes and Queries.

**The Editor is always willing to answer in this column, without any fee, all reasonable questions, as soon as possible.**

**But the following rules must be carefully observed :—**

**1. Every communication must be accompanied by the name and address of the writer.**

**2. The question must always bear upon nursing, directly or indirectly.**

**If an answer is required by letter a fee of half-a-crown must be enclosed with the note containing the inquiry.**

*Convalescent Home.*

(63) Where can I find addresses of some convalescent homes where a nurse with slight heart complaint could rest for a few weeks ?—*E. E. R.*

You will find a full list in "Burdett's Hospitals and Charities."

Will you kindly tell me if there is any convalescent home or hospital where a young lady could be received for the winter months at a very low fee. She suffers from asthma, and also has a weak heart. She could do for herself, and also help in light duties ?—*Sister.*

See reply to E. E. R. St. Mary's Home for Invalid Ladies, Dean Park, Bournemouth; the Convalescent Institution for Invalid Ladies, Erith House, Torquay; or the Royal West of England Sanatorium, Weston-super-Mare, seem suitable to the requirements of the case.

Can you kindly tell me of any invalid home in Bournemouth or Torquay where a nurse (gentlewoman) suffering from Bright's disease could be received on very reasonable terms ?—*B. E. S.*

See replies to "Sister" *re* convalescent homes.

*School Sanatorium.*

(64) Where can I get information which would guide me in setting before the authorities of a public school a statement of the alterations required in the school sanatorium necessary to bring it up to the standard required now for sick nursing ?—*School Matron.*

You could draw the attention of the authorities to defects; and their architect should then be able to deal wi h the case.

*Home.*

(65) Can you tell me of any home where an old gentleman partly paralysed could be cared for ? He is willing to pay from 10s. to 15s. weekly.—*Nurse E.*

The Hospital for Epilepsy and Paralysis, 32 Portland Terrace, Regent's Park, N.W., might receive this patient.

*Roman Catholic Probationers.*

(66) I am most anxious to become a nurse, but my being a Roman Catholic seems against me. Is there any hospital that would take me ? I should require a small salary and uniform.—*A. M. B.* and *B. E. S.*

You might apply at the West London Hospital, W. See also "The Nursing Profession: How and Where to Train," as there are many schools where all religous creeds receive due consideration.

*Nurse and Dispenser.*

(67) I am anxious to obtain a position, such as I have recently held, as nurse and dispenser to a medical man. Can I advertise in The Hospital and have my replies forwarded privately ? Will you kindly let me know the cost.—*M. I. P.*

Yes, you can advertise in The Hospital and have your replies forwarded by the Manager, to whom you must apply for information as to cost of advertisement.

*Bicycle.*

(68) 1. Would you kindly tell me what allowance a district nurse using her own bicycle might claim from her committee on that account ? 2. Where could I dispose of some false teeth to the best advantage ?—*Sister Peaceful.*

1. Lay the matter before your committee in a friendly way. 2. You can only apply to people advertising for such things. Their value is trifling.

*Training.*

(69) I am anxious to train as a nurse. What payment would I get for the first six months ?—*E. H.*

It depends upon the school which accepts you. You might get £5 or £6. See "The Nursing Profession: How and Where to Train."

*Comparative Merit.*

(70) Please tell me whether Brownlow Hill Infirmary, Liverpool, or the Hackney Union Infirmary, Homerton, offers the best training for nurses.—*Nurse Maude.*

Brownlow Hill is the larger institution, and offers a course of training in the maternity wards, which is an advantage.

*Practical Experience.*

(71) Can you tell me of any hospital, not necessarily in London, where a middle aged lady (strong) could receive three or six months' practice and experience in general nursing ? She would be willing to pay.—*C*

See regulations as to paying probationers in "The Nursing Profession: How and Where to Train." Either the London Hospital or the Middlesex Hospital might accept her.

*Health.*

(72) My health, after 16 months' training at a London hospital, broke down, owing to domestic trouble, and poor food. Could I get abroad as nurse ? Please advise me.—*C. C. B.*

It is a pity you cannot complete your training. You might try and get a situation as nurse-companion to an invalid going abroad by advertisement.

*Sanatoria.*

(73) Can you tell me of some Sanatorium in England where a nurse threatened with phthisis could go ?—*Etna.*

I have been ordered open air treatment, and I should be glad to obtain a list of sanatoria in the North of England.—*Nurse F. J.*

Are there any free, or cheap, Sanatoria for the Open-Air Treatment ? I have a delicate daughter, and am anxious for her to undergo the treatment.—*E. A.*

See "The British Sanatoria." Published by John Bale, Sons and Danielsson, Limited, Great Titchfield Street, Oxford Street, W.

*Cutting Children's Hair.*

(74) I am nurse in charge of a fever hospital. The patients are mostly children ranging in age from 3 to 12 years. Will you kindly tell me if it would be a correct thing, on my own responsibility, to cut off the whole or part of the hair from the children's heads which are dirty ? In most general hospitals and infirmaries, there is, as a rule, a barber attached, who visits on certain days, and who would, I believe, cut any patient's hair, if necessary, at the request of the ward sister or nurse in charge.—*G. H. B.*

It is best, if it can be arranged, not to have a barber going in and out. Get directions as to the cutting from the doctor in charge. He will be fully within his rights in ordering the hair to be cut.

*Dispensing.*

(75) I have had one year's training in a London hospital, and now wish to qualify as a dispenser. Will you kindly tell me the best and cheapest way to set about it ? Can I prepare at home or must I attend classes ?—*M. H.*

The best way is to become a pupil of a practical chemist. Advertisements occasionally appear in our columns for lady pupils, and the secretary, the Woman's Medical School, Handel St eet, W.C., is sometimes able to give introductions to chemists who receive such. For correspondence classes and schools see advertisements in the Nursing Section of The Hospital.

1. I should be grateful for the address of the Medical School for Women in London. 2. How can I get the post of a dispenser to a doctor ?—*A. B.*

1. See reply to *M. H.* 2. Advertise in the medical journals.

*Harmless Lunatics.*

(76) I am thinking of opening a private asylum for harmless lunatics. I have had both hospital and private experience. I hold a certificate for nursing mental disease. I want to know what is meant by a "registered" house; if I must establish my home in a town, how I can get Chancery patients, and if I must maintain a resident medical man.—*R. C.*

Write for particulars to the Secretary of the Lunacy Board of the country you wish to inhabit. London for England and Wales, Edinburgh for Scotland, and Dublin for Ireland. You can reside where you please, and must consult your own convenience as to maintaining a resident medical man. You must obtain patients, Chancery or other, through advertisements or private recommendati ns.

*District Nurse.*

(77) 1. Would it be impossible for me to get a post as district nurse, because of my age ? I am 45. 2. Can you give me the address of the Central District Nurses Home in London ?—*Mrs. S.*

Are you a certificated nurse ? If so, you should be able to get work, if not, you are too old to train. 2. This is a private home, see the post office directory.

*Address.*

The Matron of the Maternity Nursing Mission, in King's Cross Road, writes to point out that the Mission has been removed to 5 Little James Street, Bedford Row.

---

**Standard Books of Reference.**

"The Nursing Profession: How and Where to Train." 2s. net; post free 2s. 4d.

"Burdett's Official Nursing Directory." 3s. net; post free, 3s. 4d.

"Burdett's Hospitals and Charities." 5s.

"The Nurses' Dictionary of Medical Terms." 2s.

"Burdett's Series of Nursing Text-Books." 1s. each.

"A Handbook for Nurses." (Illustrated). 5s.

"Nursing: Its Theory and Practice." New Edition. 3s. 6d.

"Helps in Sickness and to Health." Fifteenth Thousand. 5s.

"The Physiological Feeding of Infants." 1s.

"The Physiological Nursery Chart." 1s.; post free, 1s. 3d.

"Hospital Expenditure: The Commissariat." 2s. 6d.

All these are published by the Scientific Press, Ltd., and may be obtained through any bookseller or direct from the publishers 28 and 29 Southampton Street, London, W.C.

## Figures and Tables

**Fig. 7. f1:**
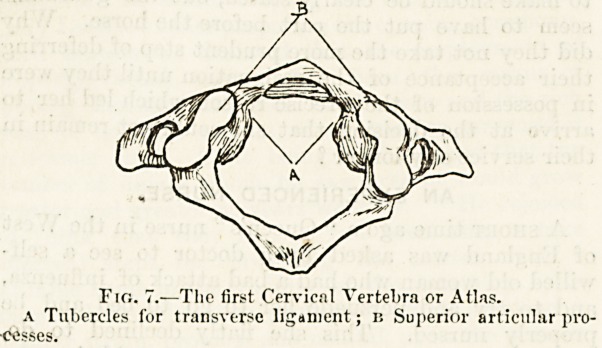


**Fig. 8. f2:**
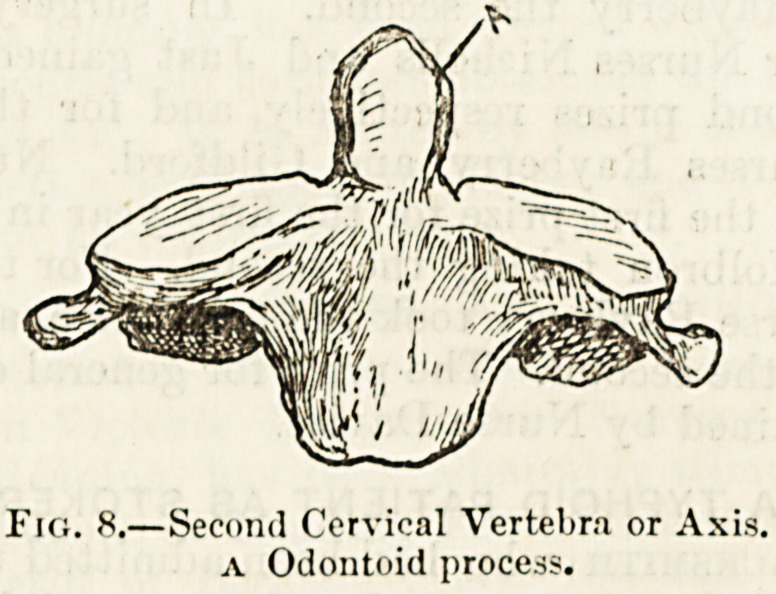


**Fig. 9. f3:**
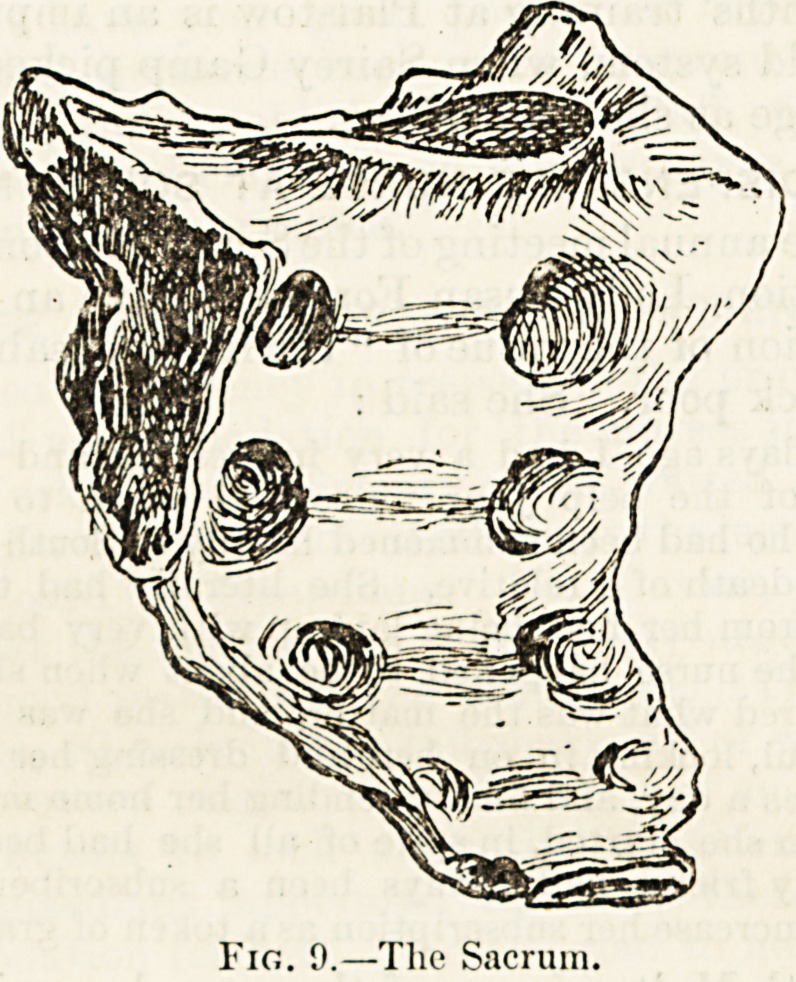


**Figure f4:**
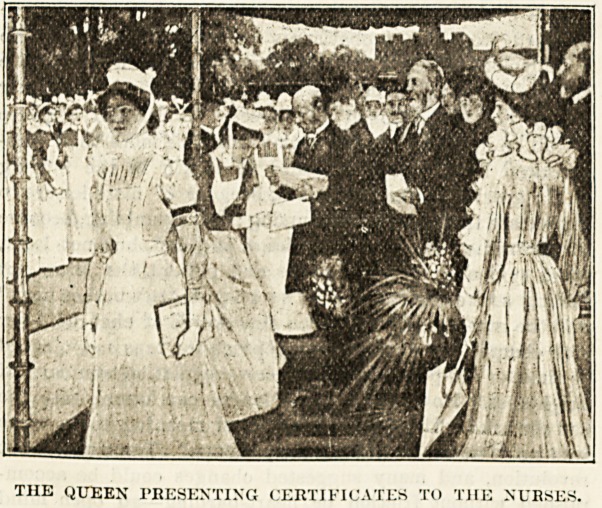


**Figure f5:**